# Sensing Hydration of Biomimetic Cell Membranes

**DOI:** 10.3390/bios11070241

**Published:** 2021-07-16

**Authors:** Madhurima Chattopadhyay, Hanna Orlikowska, Emilia Krok, Lukasz Piatkowski

**Affiliations:** Faculty of Materials Engineering and Technical Physics, Institute of Physics, Division of Molecular Physics, Poznan University of Technology, Piotrowo 3, 60-965 Poznan, Poland; hanna.orlikowska@put.poznan.pl (H.O.); emilia.krok@put.poznan.pl (E.K.)

**Keywords:** hydration, lipid mobility, lateral diffusion, FRAP, model biomembrane, solid supported lipid bilayer, biological water, hydration sensing

## Abstract

Biological membranes play a vital role in cell functioning, providing structural integrity, controlling signal transduction, and controlling the transport of various chemical species. Owing to the complex nature of biomembranes, the self-assembly of lipids in aqueous media has been utilized to develop model systems mimicking the lipid bilayer structure, paving the way to elucidate the mechanisms underlying various biological processes, as well as to develop a number of biomedical and technical applications. The hydration properties of lipid bilayers are crucial for their activity in various cellular processes. Of particular interest is the local membrane dehydration, which occurs in membrane fusion events, including neurotransmission, fertilization, and viral entry. The lack of universal technique to evaluate the local hydration state of the membrane components hampers understanding of the molecular-level mechanisms of these processes. Here, we present a new approach to quantify the hydration state of lipid bilayers. It takes advantage of the change in the lateral diffusion of lipids that depends on the number of water molecules hydrating them. Using fluorescence recovery after photobleaching technique, we applied this approach to planar single and multicomponent supported lipid bilayers. The method enables the determination of the hydration level of a biomimetic membrane down to a few water molecules per lipid.

## 1. Introduction

Phospholipid membranes are indispensable architectural components of cells, subcellular compartments, and nanometer-sized biological objects such as exosomes or viruses [1]. The basic role of biological membranes is to define boundaries and enable compartmentalization of different parts of the cell, thus fulfilling a fundamental condition for the existence of life [2]. Besides providing structural integrity, biological membranes carry out a variety of other important functions, including but not limited to, mediating and modulating the transport of ions and sugars, regulating the permeability of nonelectrolytes, and facilitating signal transduction and metabolic reactions [3,4].

Lipids, due to their amphiphilic nature, spontaneously self-assemble in the aqueous environment. This efficient self-organization has been widely used to mimic the essential lipid bilayer structures of biological systems in both basic and applied research. Bilayers in the form of black lipid membranes, vesicles (free-standing or tethered to supports), and planar supported bilayers (interacting directly with a solid substrate or tethered to the substrate) are commonly used as biomimetic membranes [5]. Such model systems, on the one hand, preserve the essential characteristic of the lipid bilayer and, on the other hand, simplify the biological membrane system so that the roles of the individual membrane components, as well as their organization and dynamics, can be effectively explored. Consequently, the use of biomimetic membranes, often integrated with functional proteins, has proven to be a powerful tool used in drug screening [6,7,8] and delivery systems [9,10], artificial cell design [11], nanoreactors [12,13], biosensors [14,15,16], and the most commercially developed water purification applications [17,18,19], to name just a few spectacular examples from the very long list reported in the literature.

Essential to the activity of biological and biomimetic membranes are their hydration properties, in which water molecules form partially ordered structures due to dipole interactions and hydrogen bonding with the membrane [20,21,22]. Of particular interest, yet remaining elusive, is the local, temporary membrane dehydration, which is believed to be one of the crucial steps in various biological processes such as adsorption of biomacromolecules or membrane fusion events. Membrane fusion constitutes a central mechanism in phenomena involving subcellular compartmentalization, cell growth, hormone secretion, neurotransmission, fertilization, viral entry, and exocytosis [1,23]. A certain amount of hydration barrier has to be overcome to initiate the hydrophobic interactions between the two fusing bilayers. To understand the molecular-level mechanism of these cellular processes, information about the local hydration state (i.e., the number of water molecules hydrating a lipid headgroup) of the membrane components at each intermediate step is very important.

For the investigation of phospholipid membrane hydration, different experimental methods have been applied. Among the most widely used, one can mention nuclear magnetic resonance spectroscopy [24], X-ray [25] and neutron [26] diffraction, dielectric relaxation spectroscopy [27,28], quartz crystal microbalance measurements [29], linear and nonlinear infrared spectroscopy [30,31], and various fluorescence microscopy approaches [32,33,34], often combined with the theoretical simulations [22,35]. In particular, fluorescence methods are convenient tools for studying lipid membranes due to the existence of the ever-expanding palette of fluorescent probes and techniques that allow for data collection over a wide range of spatial and temporal scales. Fluorescence studies aimed at understanding the local hydration state of the lipid membrane rely on dedicated fluorophores, whose spectroscopic and photophysical properties depend on the microenvironment (so-called environment-sensitive probes), and in particular, on the polarity of the immediate environment in which they are located [33]. As such, various fluorescence-based techniques have flourished such as fluorescent solvent relaxation [36,37,38], general polarization [39], and red edge excitation shift [40,41]. Although these techniques provide important information about the lipid bilayer hydration, they pose two major inconveniences, namely the need to know the precise probe location and the challenging process of data correlation, both crucial for drawing valuable conclusions [42,43]. Consequently, it is clear that a novel experimental approach towards molecular-level sensing of local hydration conditions of lipid membranes is needed.

Here, we present a new approach to gain insight into the hydration state of the lipid membranes. It is based on measuring the change in the lateral diffusion coefficient of lipids forming a bilayer, which is dependent on the number of water molecules hydrating the lipids. We illustrate and validate the method using fluorescence recovery after photobleaching (FRAP) technique applied to a planar solid-supported lipid bilayer (SLB). Our approach exhibits sensitivity at the quasi-single molecule level, enabling the determination of the hydration level of a biomimetic membrane down to a few water molecules per lipid. This new approach has the potential to reveal the local heterogeneity in hydration of biomimetic and biological lipid membranes, and thus sheds light on the processes incorporating changes in membrane hydration, such as membrane fusion.

## 2. Materials and Methods

### 2.1. Materials

1,2-dimyristoleoyl-sn-glycero-3-phosphocholine (DMoPC), egg yolk sphingomyelin (SM), and cholesterol were purchased from Avanti Polar Lipids (Alabaster, AL, USA). 1,2-dioleoyl-sn-glycero-3-phosphoethanolamine (DOPE) labeled with Atto 633, sodium chloride (NaCl), and chloroform (HPLC grade) were purchased from Merck KGaA (Darmstadt, Germany). 4-(2-hydroxyethyl)piperazine-1-ethanesulphonic acid (HEPES PUFFERAN^®^) was obtained from Carl Roth GmbH&Co KG (Karlsruhe, Germany). Calcium chloride (CaCl_2_) was purchased from P.P.H. STANLAB sp. j., Lublin, Poland. Optical adhesive glue Norland 68 was purchased from Thorlabs Sweden AB (Mölndal, Sweden). All the materials were used without further purification. The ultrapure water (ddH_2_O) was obtained by using Milli-Q reference water purification system from Merck KGaA (Darmstadt, Germany).

### 2.2. Methods

#### 2.2.1. SLB Preparation

SLBs were prepared by a widely used vesicle deposition method [44] with required modification. For single component SLBs, 10 mM solution of DMoPC was prepared in chloroform along with 0.1 mol% of DOPE-Atto 633. DMoPC, egg SM, and cholesterol were mixed in 1:1:1 molar ratio with an overall 10 mM lipid concentration, along with 0.1 mol% of Atto-633-DOPE, for the preparation of phase-separated SLBs. The chloroform was evaporated by dry nitrogen gas depositing a thin film of lipid at the bottom of the glass vial followed by vacuum desiccation for a minimum of 2 h. After complete evaporation of the organic solvent, a 10 mM suspension of DMoPC multilamellar vesicles (MLVs) was prepared by dissolving the lipid film in a suitable amount of 10 mM HEPES with 150 mM NaCl buffer, adjusted to pH 7.4. The solution was vigorously vortexed and heated to 60 °C to obtain a homogeneous suspension of MLVs. Aliquots of 1 mM lipid concentration were prepared by diluting the solution 10 times with the previously mentioned buffer solution and stored at −20 °C in sterilized glass vials for further use. In order to obtain small unilamellar vesicles (SUVs) from MLVs, the aliquoted lipid solution was bath-sonicated for at least 10 min until the solution became clear. A thin sheet of freshly cleaved mica was glued to a coverslip using UV-activated glue Norland 68. A half-cut Eppendorf tube was attached and sealed with silicone adhesive on top of the coverslip. At room temperature, 100 µL of 1 mM SUVs solution was deposited on mica followed by immediate addition of 0.2 µL of 0.1 M CaCl_2_ in a buffer. After 30 s, 400 µL of HEPES-NaCl buffer (pH adjusted to 7.4) was added to the sample to prevent drying out of the SLB. The sample was incubated for 30 min at room temperature and then rinsed multiple times with a total of 20 mL of buffer solution. To obtain a fully hydrated sample, the Eppendorf tube sample reservoir was completely filled with buffer, closed with another coverslip, and sealed by silicone adhesive.

#### 2.2.2. SLBs Hydration State Control

Precise control over the hydration state of the sample was achieved using a home-built humidity control set-up. Nitrogen gas of desired relative humidity (RH) was purged inside the open half-cut Eppendorf tube of the sample. The relative humidity of the nitrogen gas was regulated by mixing wet (~90% RH) and dry (2%–3% RH) nitrogen with a suitable ratio. The flows of wet and dry N_2_ gas were individually adjusted using two manual valves while monitoring the readings shown in the two flowmeters connected to the wet and dry N_2_ flow paths. A third flowmeter along with a manual valve were used to keep the final flow of N_2_ gas constant at ~1.2 L/min throughout the experiment. An electronic hygrometer (0%–95% RH range and 1% precision) was employed to record the RH and temperature of the final flow purged towards the sample, and it also created a feedback loop for adjusting the amount of wet and dry N_2_ gas. The silicone seal of the fully hydrated sample was cut and the buffer was pipetted out completely followed by immediate purging of N_2_ gas of 90% RH. FRAP measurements were taken for SLBs equilibrated to 90%, 70%, 50%, 30%, and 0% RH at constant temperature. The dehydration and rehydration were performed in steps of ~20% RH with change rate of 2%–3% RH/min. The sample was rehydrated in a similar manner that is by purging N_2_ gas and increasing its RH. Finally the sample was again filled with buffer and sealed to obtain bulk rehydration of the sample.

#### 2.2.3. FRAP Experiments

Zeiss LSM 710 (Carl Zeiss, Jena, Germany) microscope with 40×, 1.3 NA oil immersion objective was used for confocal imaging and FRAP experiments. Atto-633 was excited by a laser with a 633 nm wavelength. Laser power was adjusted to a minimum to avoid excessive overall photobleaching of the sample during measurements. For FRAP experiments, a selected circular spot of 10 µm diameter within a 50 µm × 50 µm area was instantaneously bleached with maximum laser power in every measurement. In the experiment 100 consecutive fluorescence images of the same area were recorded with 0.5 s intervals. The data were analyzed using MATLAB software and the diffusion coefficient was determined considering free Brownian lateral diffusion of lipid molecules in the membrane, by fitting the fluorescence recovery curve using modified Soumpasis Formula (1) [45]:

(1) F(t)=b+a·f(t),

where *a* is the amplitude of the recovery function, *b* is the remaining fluorescence after bleaching, and *f*(*t*) is the Soumpasis function. The FRAP traces were normalized taking the overall fluorescence intensity signal of the image except the bleached spot as the reference. FRAP experiments were performed on one representative single component and one phase-separated SLB throughout complete dehydration-rehydration cycles. A minimum of 5 FRAP measurements at various areas of the samples were recorded at a particular RH for each sample.

## 3. Results

### 3.1. Measurement and Analysis of Diffusion Coefficient of Lipids at Different Hydration Conditions

In this study, lateral diffusion of DMoPC in single component SLB as well as in phase-separated DMoPC/egg SM/cholesterol 1:1:1 SLB was investigated by FRAP experiments at different hydration conditions, starting from fully hydrated to membrane equilibrated to ~0% RH. In order to achieve the desired hydration state, the SLBs, immediately after removal of bulk water from the sample, were carefully equilibrated to the atmosphere of a nitrogen gas of specific relative humidity using our home-built hydration control unit. SLBs dehydrated by this method can withstand multiple dehydration-rehydration cycles without experiencing major structural damage [46].

**Figure 1:**
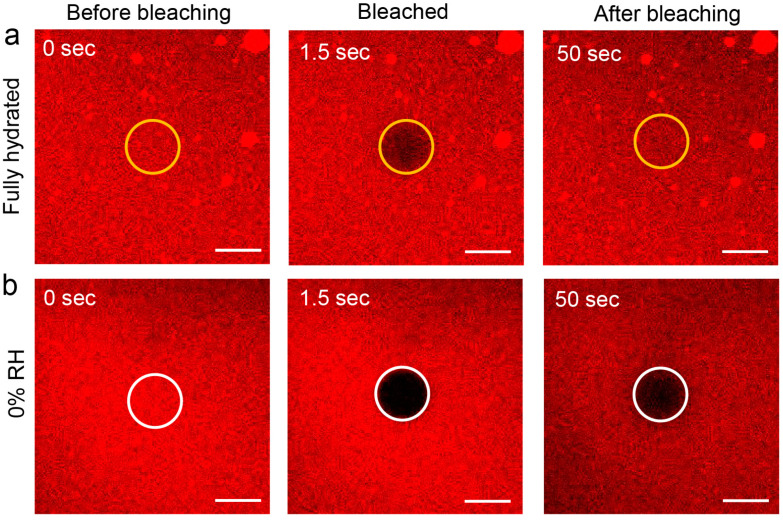
Representative confocal images from the FRAP experiment for a fully hydrated, single component SLB (**a**) and SLB exposed to 0% RH (**b**). The areas marked by yellow (**a**) and white (**b**) circles denote the spot of 10 µm diameter that was bleached during FRAP measurements. In case of fully hydrated sample (**a**), the bleached area completely regained its fluorescence within 50 s, while for sample exposed to 0% RH (**b**), the spot remained largely bleached after 50 s. The scale bars correspond to 10 µm.

**Figure 2:**
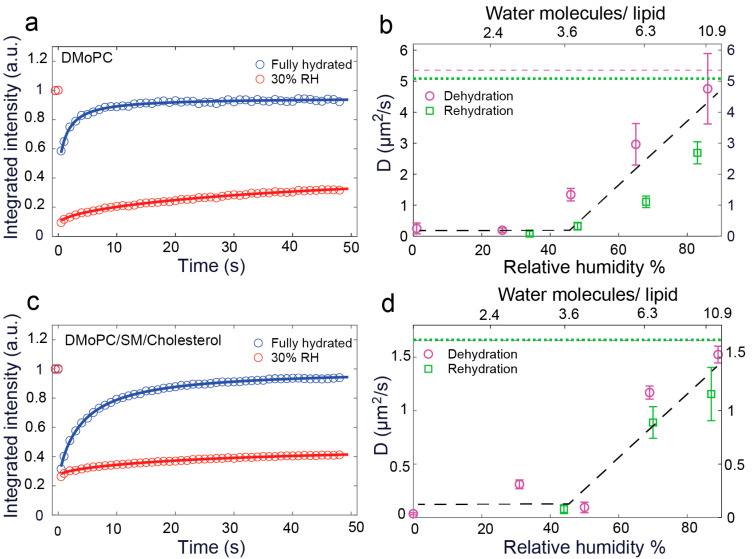
FRAP traces of fully hydrated and dehydrated down to 30% RH SLBs for a single component (**a**) and phase-separated (**c**) membrane. Panels (**b**,**d**) show the dependence of diffusion coefficient with RH for a single component and phase-separated SLB, respectively. The magenta dashed and green dotted lines are the average values of D at the fully hydrated condition and fully rehydrated condition, respectively. The black dashed line is a guide to the eye to highlight the trend in D with de(re)hydration.

The representative confocal images, selected from the FRAP measurement series of single component SLB at pre-bleached, bleached, and recovered (completely or partially) conditions are shown in Figure 1. Clearly, remarkable differences in bleaching depth and recovery of fluorescence intensity within the bleached spot are observed. FRAP experiments are based on measuring the recovery of fluorescence intensity in the bleached area that is caused by lateral Brownian motion due to the reorganization of bleached and non-bleached lipid molecules [45]. The dehydrated membrane showed higher bleaching depth and slower fluorescence intensity recovery (Figure 1b) than a fully hydrated one (Figure 1a). Thus, it can be concluded that there is a vivid decline in lateral mobility of lipids in the absence of full hydration of membrane constituents. This observation is clearly reflected in the FRAP traces for fully hydrated and dehydrated (~30% RH) membranes, as shown in Figure 2a,c, for single component and phase-separated SLBs, respectively. In the case of fully hydrated membranes, the fluorescence intensity recovers up to 95 ± 2% of the initial intensity within ~50 s after bleaching, whereas more than 50% fluorescence recovery is scarcely achieved for dried SLBs within the same time frame. For a detailed investigation of the dependence of the lateral diffusion (D) of lipids on hydration conditions, diffusion coefficients of DMoPC were measured for single component and phase-separated SLBs at various relative humidity levels, starting from fully hydrated to ~0% RH via 85%, 65%, 45%, and 30% RH. The SLBs were rehydrated subsequently by increasing RH levels in steps up to ~85%, and they were finally rehydrated fully with the addition of bulk water. Figure 2b,d depicts the diffusion coefficients of DMoPC during a complete dehydration and rehydration cycle for single component and phase-separated SLBs, respectively, averaged over at least five FRAP traces from various spots of the sample at a particular RH level. It should be duly noted that the absolute mobility of liquid-disordered (L_d_) phase lipids in phase-separated membranes is significantly lower than in pure L_d_ phase, single component membranes, as part of L_o_ lipids and cholesterol partition into the L_d_ phase, thus increasing the order and viscosity [47].

At the fully hydrated condition, the diffusion coefficients of DMoPC in a single component and phase-separated SLBs were found to be 5.35 ± 0.79 µm^2^/s and 1.66 ± 0.22 µm^2^/s, respectively. A roughly 3.5-fold higher D explains the shallower bleaching of fully hydrated single component SLB compared to the phase-separated one (see Figure 2a,c). In spite of the difference in absolute values of diffusion coefficients of lipids for both systems, the change of lateral diffusion followed an analogous trend with dehydration and subsequent rehydration. A strong correlation between the lipid mobility and the relative humidity of the sample environment was observed. The D of DMoPC in the single component SLB steadily dropped down from 4.75 ± 1.14 µm^2^/s to 0.25 ± 0.18 µm^2^/s as the hydration of the atmosphere was lowered from ~85% RH to ~0% RH. Likewise, in phase-separated SLBs, a sharp downfall of D was noticed from 1.52 ± 0.08 µm^2^/s at ~85% RH to 0.04 ± 0.01 µm^2^/s at ~0% RH. This firmly indicates that water molecules attached to the polar head group of the lipid molecules play a key role in modulating the lateral movement of lipids. Interestingly, with consecutive rehydration, lipid mobility increased back following the same trend as during dehydration, with relatively lower absolute values of D. However, with bulk rehydration, the lipids regained their natural mobility, which is the same as for fully hydrated conditions measured before de- and rehydration. Thus, from fully hydrated to fully rehydrated conditions, D changes reversibly with change in native hydration state of the membrane. The strong dependence of D on the membranes’ hydration state can be associated with a reversible change in the local hydration structure around the lipid head group caused by de(re)hydration [46]. Furthermore, it is clearly evident from Figure 2b,d that the most dramatic change of D values took place in the range of 100–50% RH; lowering the relative humidity below this range hardly affected the lateral diffusion of lipids. This breaking point at ~50% RH can be correlated with the breaking of clathrate cage structure around the phosphocholine group of DMoPC molecule [46]. We note that at the salt concentrations used in the buffer for experiments (10 mM HEPES and 150 mM NaCl), lipid diffusion remained unaffected by the change in ionic strength [48].

The presented results reveal that the lateral diffusion of lipids in the L_d_ phase of the zwitterionic lipid bilayer is particularly sensitive towards the hydration state of the membrane, or more precisely, of the lipid itself. Moreover, the dependence of D on the hydration of the membrane constituents uniformly applies to phosphatidycholines (PC) in a completely liquid-disordered environment as well as in phase-separated membranes, irrespective of the presence of L_o_ domains slowing down the overall mobility.

### 3.2. Hydration Sensing

**Figure 3:**
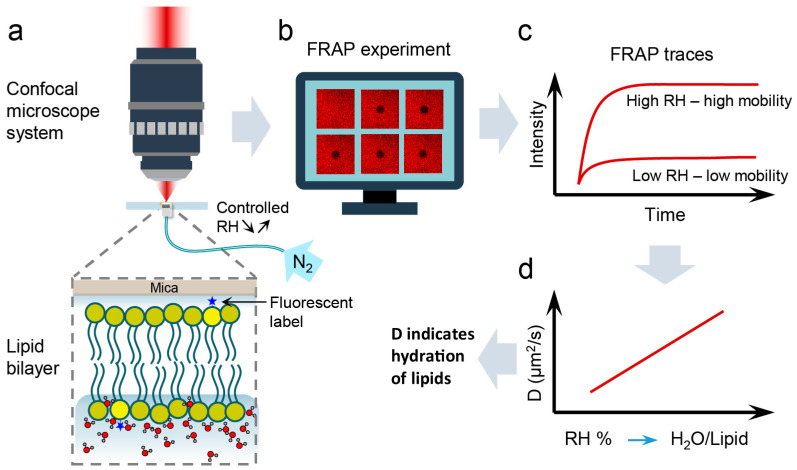
Schematic diagram of the hydration sensing concept. (**a**) Once the bulk water is removed, sample is inserted into the microscope system and subjected to a controlled dehydration/rehydration process using nitrogen gas of known humidity. (**b**) At each hydration state, FRAP measurements are performed. (**c**) From FRAP traces acquired for a particular humidity, the diffusion coefficient (D) of lipids is calculated. (**d**) The diffusion coefficient is then presented as a function of relative humidity (RH) of the sample environment, which in turn can be directly related to the number of water molecules hydrating a single lipid molecule. Thus, using this calibration plot, the hydration of the lipid membrane can be determined from the measured lipid lateral diffusion coefficient.

Our results reveal a significant dependence of the diffusion coefficient of lipids on their local hydration state. Thus, it gives the possibility to be exploited as a technique to quantify the hydration level of the membrane by using D as a hydration indicator. In previous X-ray diffraction [49] and infrared spectroscopy [30] studies, the RH of the membrane environment has been correlated with the number of water molecules present in the hydration shell of a single lipid molecule, specifically in its head group region. Approximately 10.5, 6.3, 3.6, and 2.4 water molecules, averaged from the results of the above-mentioned experimental studies, were found per PC lipid when stacked lipid membranes were exposed to an atmosphere with 95%, 75%, 50%, and 25% RH, respectively. Marrying these findings with our results, the number of water molecules per lipid head group corresponding to a particular RH can be correlated with D. Consequently, the precise determination of the lipids’ diffusion coefficient can give insights into the local hydration state of the membrane. The changes in the lipids’ mobility can be used as a local hydration sensor not only in biomimetic membranes, but also in native cell membranes. The general concept of our approach is depicted in Figure 3. It should be noted here that while extrapolating the above experimental results for membranes equilibrated with 0% RH, one would expect the membranes to be free of water. However, previous molecular dynamics simulations revealed, even after drastic drying, up to 3–4 water molecules per lipid remain strongly hydrogen bonded to the carbonyl and/or phosphate group [22,35]. Hence, for membranes equilibrated to RH < 25%, one can state that there are not more than four water molecules associated with the lipids.

### 3.3. Validation of Hydration Sensing Approach

**Figure 4:**
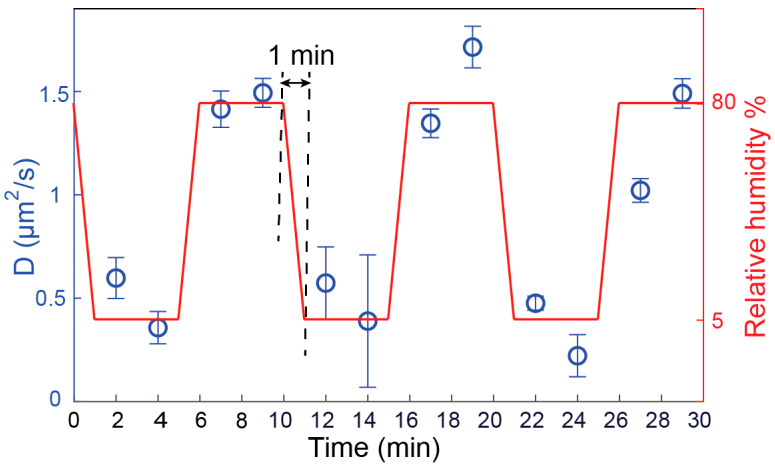
Diffusion coefficients of single component DMoPC SLB exposed to a successive switching between high (80%) and low (5%) RH over time. Each data point corresponds to the diffusion coefficient derived from a single FRAP trace. The confidence bounds of the fits of the FRAP traces were used to form the error bars for the data points. One minute time was taken to alter the RH from 80% to 5%. In the following four minutes, two FRAP measurements were performed before re-altering the RH.

The reversibility, repeatability, and response time of the sensor are crucial criteria to validate a sensing technique. To check these parameters, a single component DMoPC SLB was exposed to high (80%) and low (5%) RH alternatively several times. The RH was instantaneously adjusted from 80% to 5% by closing the N_2_ flow through the water reservoir and only letting dry N_2_ flow over the membrane. A humidity sensor attached to the flow tube close to the outlet was used to continuously monitor the RH of N_2_ gas. The set RH (5% or 80%) was reached typically within 1 min. Subsequently, the RH was kept constant for the next four minutes after each consecutive RH alteration, and two FRAP measurements were performed. Figure 4 shows the change of D over time upon sudden increase or decrease in RH in several dehydration-rehydration cycles. Clearly, the successive de(re)hydration cycles do not leave a permanent effect on the lateral diffusion of lipids, instead, a variation of D is absolutely reversible and repeatable. We note that the measured D at high/low RH appeared to be lower/higher than the average D values at these two hydration levels, as shown in Figure 2b. This results from the fact that the RH of the purged nitrogen gas was swapped quickly (75% RH change in 1 min), and the FRAP measurements were started immediately before the SLB was fully equilibrated to the particular RH provided. This is also evident from the fact that the second FRAP trace always yields higher (for 80% RH) and lower (for 5% RH) D, while the membrane still undergoes equilibration. Generally, in our experiments, we noticed that the equilibration time at a particular RH was around 10 min. Nevertheless, the prompt change of D, shown in Figure 4, within 1 min of changing RH is sufficient to unambiguously indicate a significant change in the hydration state of the system. This confirms that a quick response in D is expected as soon as the hydration state is altered.

## 4. Discussion

### 4.1. Applicability and Measurement Criteria

Transient, local dehydration of membranes is one of the most important intermediate steps in numerous biological processes, for example, endo- and exocytosis, fertilization, viral entry, biogenesis of muscle tissue [50], etc. The central mechanism of these processes constitutes membrane fusion. The lipid layers merge, overcoming a specific hydration barrier. This results from the removal of water molecules from lipid head groups when the hydrophobic tails come into contact with the water-caged hydrophilic head groups of the two lipid layers [51]. A molecular-level understanding of such processes requires quantification of the local hydration state in terms of the number of water molecules attached per lipid in each intermediate step of the process. Our hydration sensing methodology forms a solid base for developing a complete stepwise map of hydration structures of lipid molecules in the course of membrane fusion or any other process that involves local dehydration of the membrane. Here, we verified the hydration sensing approach for DMoPC as a representative PC lipid, being one of the most abundant group of lipids found in biological cell membranes. As water molecules present in the clathrate cage around phosphocholine head group are related to lipid mobility, it is expected for other PC lipids to show dependence of the diffusion on hydration. It is important to note that the observed correlation between the diffusion coefficient and hydration state of the membrane is equally valid for a single component membrane as well as for more complex, multicomponent lipid bilayers that exhibit phase separation and formation of domains.

A few criteria of measurement conditions should be considered for an effective and fruitful hydration sensing. Apart from the hydration level, several additional parameters affect lipid dynamics in a membrane. The presence of saturated and unsaturated lipids, cholesterol, membrane proteins, and phase separation in membranes influence the absolute values of the diffusion coefficients of lipids. In presence of various ions in the medium, the complex formation of an ion binding with more than one lipid may slow down the mobility of lipids and weaken the polarization of water molecules in the interior of the membranes [48]. Moreover, physical factors such as temperature variation also affect the diffusion of lipids. In order to obtain a clear picture of the hydration state of lipids in a membrane, lipid diffusion should be measured keeping all other parameters constant. In complex biological membranes, where a variety of parameters modulates lipid dynamics, the presented approach still enables comparative studies of hydration structure and hydration heterogeneity between various membrane sites. The high sensitivity of this methodology (few water molecules per lipid) allows for the qualitative determination of the hydration state.

### 4.2. Perspectives

From the technical standpoint, it must be emphasized that our approach is not limited to using the FRAP technique. In fact, it could be easily transferred to any method that enables quantitative analysis of lipid diffusion, such as, for instance, fluorescence correlation spectroscopy (FCS). Although both FRAP and FCS techniques meet the temporal resolution needed to resolve membrane dynamics, their spatial resolution, when performed with conventional confocal microscopes, is diffraction limited [52]. Consequently, the heterogeneity of diffusion, and thus the heterogeneity of hydration that can occur at the nanoscale, may be overlooked due to the inherent ensemble averaging across the entire illumination area [53,54]. One of the strategies to breach the diffraction limit barrier is a coupling of the well-established FCS approach with super-resolution imaging technique such as stimulated emission depletion (STED) nanoscopy [52,55,56]. STED-FCS has been successfully applied to model biomembranes for studying nanoscale lipid dynamical heterogeneities induced by pore-forming proteins [56], as well as to the plasma membrane of living cells to discern nanoscale molecular diffusion modes [52]. An alternative approach is marrying the aforementioned FSC with near-field scanning optical microscopy, capable of confining the illumination light at the nanoscale [54,57]. High spatial resolution has also been achieved with a single-molecule FRAP (smFRAP) approach, which was employed to determine the distribution and translocation rates of inner and outer nuclear membrane proteins in live cells in real-time conditions [58]. An alternative solution is to trace the motion of individual particles attached to the lipids of interest using the single-particle tracking (SPT) technique [59]. Although conventional SPT yields a limited temporal resolution (often in the millisecond range), solutions have already emerged to improve it by combining SPT with interferometric scattering microscopy (iSCAT) techniques. Using the SPT–iSCAT approach, microsecond temporal resolution with simultaneous sub-1 nm spatial precision of lipid localization has been achieved [60,61,62].

Clearly, the possibilities are tremendous. Synthesizing the evidence from our studies with single-molecule approaches could truly provide capabilities to sense biomimetic and biological membrane hydration at the single-molecule level. Adsorption of large biomolecules (such as proteins) onto the membrane often disturbs the hydration layer locally in the vicinity of the binding site [63]. Using single-molecule approach, it is feasible to sense hydration changes even in the case of single biomolecule–membrane interactions.

For using lipid diffusion as a hydration marker, two distinct approaches can be undertaken. One of these approaches is to provide an absolute number of water molecules per lipid in a membrane looking at the diffusion coefficient of the very lipid. However, as the exact number of water molecules per lipid varies depending on the lipid structure and its surroundings, the number of water molecules per lipid at different relative humidity levels should be determined precisely for that specific system. Additionally, to gain information on the number of water molecules correlated with diffusion coefficient of lipids accurately, calibration for complete dehydration and rehydrated cycle is essential. On the other hand, for a system that is homogeneous in terms of diffusion-affecting parameters other than hydration state, by local probing of lipid diffusion in different sites of interest, a qualitative comparison of the hydration structure and state can be revealed. In this scenario, the method becomes self-referential and the requirement for calibration is eliminated.

## 5. Conclusions

We developed a novel approach for sensing the hydration state of lipids and hydration heterogeneity within lipid membranes, based on the strong correlation between lateral mobility of phospholipids with the number of water molecules forming the hydration shell around lipids’ polar head groups. Hence, the diffusion coefficient of a lipid can be considered as a measure of its hydration state at a molecular level. The change in lateral diffusion coefficients of lipids with the number of water molecules hydrating a lipid is fully reversible and repeatable. For a comparative study of hydration heterogeneity of different sites within the membrane, the methodology is self-sufficient and self-referential. Finally, the presented approach can readily be equipped with a single-molecule hydration sensing capabilities if the diffusion coefficient is measured with single-molecule sensitivity approaches such as SPT or FCS. Thus, the approach for hydration sensing possesses an enormous potential for quantitative, molecular-level studies of the (de)hydration-mediated processes in lipid membranes.

## 6. Patents

Chattopadhyay, M.; Krok, E.; Orlikowska, H.; Piatkowski, L. Method for Measuring the Local Hydration of Lipid Layers of Biomimetic and Biological Systems. Patent Application (Poland), P.437600 and P.437601, 16.04.2021.

## Data Availability

All data underlying the study are available from the authors.
